# The Importance of Testing the Quality and Authenticity of Food Products: The Example of Honey

**DOI:** 10.3390/foods12173210

**Published:** 2023-08-25

**Authors:** Natalia Żak, Aleksandra Wilczyńska

**Affiliations:** Department of Quality Management, Gdynia Maritime University, ul. Morska 81-87, 81-225 Gdynia, Poland; a.wilczynska@wznj.umg.edu.pl

**Keywords:** quality and authenticity assessment, honey

## Abstract

The aim of this study was to review methods of honey testing in the assessment of its quality and authenticity. The quality of honey, like other food products, is multidimensional. This quality can be assessed not only on the basis of the characteristics evaluated by the consumer during purchase and consumption, but also on the basis of various physicochemical parameters. A number of research methods are used to verify the quality of honeys and to confirm their authenticity. Obligatory methods of assessing the quality of honey are usually described in legal acts. On the other hand, other, non-normative methods of honey quality assessment are used worldwide; they can be used to determine not only the elementary chemical composition of individual types of honey, but also the biological activity of honey and its components. However, so far, there has been no systematization of these methods together with a discussion of problems encountered when determining the authenticity of honeys. Therefore, the aim of our study was to collect information on the methods of assessing the quality and authenticity of honeys, and to identify the problems that occur during this assessment. As a result, a tabular summary of various research methods was created.

## 1. Honey Quality and Authenticity

Many attempts have been made to define food quality. One of them is based on the theory of a multidimensional set of features that can be objective (measurable, taking into account research results) or subjective (immeasurable, taking into account the opinion of the consumer) [1,2]. A universal definition of quality has been proposed in an international standard on quality management: “Quality is the degree to which a set of inherent properties meets the requirements” [3]. This definition can also be applied to food, and the requirements mentioned here can be both requirements contained in legal acts and consumer requirements. Food product quality is a concept that corresponds to a set of many attributes (e.g., product specific features, product safety, acceptance by the consumer). One of the attributes of food quality is its authenticity. Food authentication and traceability are current topics in the food sector since they enable food quality and safety control [4,5]. According to researchers, the authenticity of the product is understood as confirmation of the requirements for ensuring quality, composition, safety, usability, brand, and origin along with the information/declaration provided to the consumer by the manufacturer. It determines whether the product is really what the manufacturer declared [6,7,8,9,10]. The global definition of food authentication is problematic. There is no clear definition of this concept in legal acts covering the US, EU countries and cooperating countries. The definition of food authenticity is also not included in the Codex Alimentarius, which introduces and promotes definitions and requirements for food that facilitate the harmonization of international food circulation. Only the definition of contamination is formulated here, but there is no definition of authenticity or the adulteration of food [11].

The definition of authenticity has evolved over time and with the development of production, research infrastructure and research. Initially, this phenomenon was associated only with food counterfeiting and misleading the consumer. The composition of the product was changed without informing consumers. For example, more valuable ingredients were replaced with less valuable ones. The counterfeit product resembled the original product, but its quality was lower [12]. Further actions violating the authenticity of the products and intentionally misleading the consumer concerned their improper labeling. For example, terms such as “bio”, “eco”, “protected designation of origin” and “protected geographical indication” were used unlawfully without identifying the origin of the raw materials [5,8,13,14].

In the case of food, it is much more often said that it is adulterated than authentic. According to Spink and Moyer [15], food adulteration is: “A collective term that includes knowingly and intentionally substituting, adding, tampering with, or misrepresenting food, food ingredients, or food packaging: false or misleading claims about a product for economic gain”. However, according to Everestine et al. [16], “food is adulterated intentionally for financial gain”.

It is also a great challenge to clearly define the quality and authenticity of products, taking into account their multi-criteria parameters. For this, fast and reliable methods must be available, which will be supported by specific and reliable markers. All this is aimed at withdrawing counterfeit products from the market, but also at preventing similar accidents [17]. Below, Table 1 presents the factors that define the quality and authenticity of food products and the methods of their assessment.

The evaluation of the quality of food products through the performance of a series of analyses and tests is a requirement resulting from legal acts. The main purpose of these rules is to ensure food safety. In addition, food products must meet the requirements of consumers and companies [21,22]. The quality of food products is also treated as an element of marketing, competitiveness and company prestige, which translates into an increase in profits [12].

Being a natural product, honey is also considered to be one of the most frequently adulterated products. Therefore, issues related to ensuring its quality and safety have put it at the forefront of the mind of global trading concerns and food regulatory agencies [23]. The literature gives the opportunity to indicate many practices used by beekeepers and honey producers that distort the authenticity of honey, such as the following:

- Mixing honey with water and sugar or selling solutions of water, sugar and flour, and boiled flowers;
- Mixing varieties;
- The sale of imported honeys (often of lower quality, not meeting the requirements as to the composition and properties) or their mixture with domestic honeys;
- The addition of imported honeys containing residues of drugs prohibited in EU countries due to their toxic effects (e.g., chloramphenicol—an antibiotic found in honeys from China);
- Placing incorrect data on the botanical and geographic origin of the product;
- Added sugar syrups (glucose–fructose);
- The addition of potato and beetroot syrup;
- The addition of molasses;
- Adding inverts to honey in order to increase its commercial weight and achieve quick profits (an illegal practice and foreign to beekeeping ethics);
- Feeding bees with sugar during the nectar period of plants;
- The repeated heating of honey in order to decrystallize it;
- Harvesting honey before its maturity;
- The overuse of veterinary drugs and antibiotics [12,23,24,25,26,27,28,29,30,31,32].

Honey is subject to the general requirements of the EU and national legislation. Presently, obligatory quality requirements for Polish honey are specified in the Resolution of the Ministry of Agriculture and Rural Development, dated 3 October 2003, regarding the detailed requirements for the commercial quality of honey. The above resolution basically corresponds with the requirements of the Worldwide Standard for honey, developed and approved by the Commission of Food Code from 2001 (Codex Alimentarius: Draft revised standard for honey 2001) [11], and to the European Directive for honey [33]. Evaluation of the quality of honey in accordance with those standards includes determining its organoleptic characteristics, distinguishing dominant pollens and indicating its basic physicochemical parameters (moisture, electrical conductivity, 5-hydroxymethyl furfural content, apparent reducing sugars, apparent sucrose, insoluble matter and diastase activity). Table 2 presents limit values for individual physicochemical parameters and an interpretations of their excess.

Honeys adulterated with sugar syrup or other inverts may contain starch dextrins and the wrong profile of sugars. An analysis of the literature showed that a reduced level of electrical conductivity and diastase number, while increasing the sucrose content, may indicate the deliberate adulteration of honey with sugar syrup [53]. Too-low values of such parameters as ash content, enzyme activity or proline content may mean adulteration of honey with sugars [51]. 

**Figure 1:**
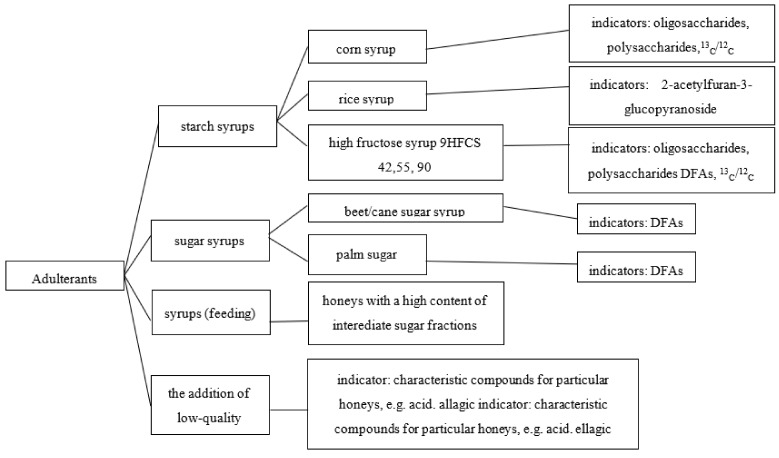
The most common substances added to natural honey for the purpose of adulterating it and indicators allowing for their detection. Source: [54].

The quality and quantity determination of the basic components of honey does not pose considerable difficulties because it is accomplished using simple analytical techniques. But these parameters do not allow for a precise determination of the geographical or botanical origin of the honeys. In addition to assessing the quality of the physicochemical parameters, Kowalski and Łukasiewicz indicated the most common substances added to natural honey for the purpose of adulterating it and indicators allowing for their detection [54] (Figure 1). 

The concepts of quality and the authenticity of honey are interpenetrating notions, because authenticity is one of the attributes of quality. In the case of testing the quality and authenticity of honey, the tests performed can be divided into a number of methodological groups resulting from legal acts [27,28,29,30,31,32,33,34,55] and based on modern methods used by numerous research teams. Therefore, the aim of this study was to determine the importance of the food product testing factor in assessing the quality and authenticity of honey and the systematization of this information. 

## 2. A Review of the Methods for Assessing the Quality and Authenticity of Honeys

The selection of the appropriate research method and interpretation of the results of quality parameters in terms of confirming the authenticity of honey is not easy. It should be remembered that honey is a unique product whose quality is influenced by many parameters. In connection with the above, a review of these methods was created, from simple methods to more advanced methods. Table 3 presents problem identification factors and groups of honey authenticity problems, with the aim of facilitating the selection of research methods. In addition, methodological limitations are indicated for each method.

The research material consisted of original research articles in the field of the quality and authenticity assessment of varietal honeys, which were published up to the first half of 2023, totaling more than 160 scientific articles. The search for the articles was carried out by entering key words, such as honey, honey authenticity, geographical origin, honey quality tests, honey storage, honey color, pollen analysis, 5-HMF, diastase number, antioxidants, spectroscopy and fluorescence. A search of publications from the last 10 years was performed, but sometimes older works were included. The condition for using older publications was the well-founded knowledge contained in them. Research databases were searched, such as Google Scholar, Science Direct, MDPI, Knovel, Applied Science & Technology Source, Scopus, Taylor and Francis online and Ebsco host.

Based on the analysis of the literature, 16 methodological groups were distinguished for research in the field of honey quality and authenticity—Table 3. Below the table, there are comments on the limitations and disadvantages of the use of individual methods.

### 2.1. Melissopalynological Analysis (Honey Pollen Analysis)

Quantitative analysis consists of counting all plant parts (N), i.e., pollen grains, fungal spores and algae hyphae, yeast, starch grains and others in 10 g of honey. It allows honey to be assigned to one of five classes.

The qualitative analysis determines the varieties of honey, with particular emphasis on honey and their additives from other climatic zones. It is the basis for the determination and classification of the nectar plants involved in the production of honey. It consists of counting pollen grains in a microscope preparation, and then comparing them with the provisions regulating the content of guiding pollen in varietal honeys.

The minimum percentages of guiding pollen for honeys are as follows: rapeseed—45%, acacia—30%, linden—20%, buckwheat—45%, heather—45%, and polyfloral—none.

This method is a classic approach to confirming the botanical origin of honey. It is useful in the control and classification of honeys of individual varieties and those imported from different regions of the world. However, it is a time-consuming method and depends on the expert’s experience. This method also allows nectar honey to be distinguished from honeydew [26,55,56,57,58,59,60,61,62,63,64,65].

It is based on the assumption that certain types of pollen are present in a given area, which makes it possible to determine the origin of honey on this basis [65].

### 2.2. Sensory Analysis

The bases for this form of research are the senses and feelings related to the smell, taste, color, appearance, and consistency of the product. This is a characterization analysis for honey varieties and their geographical origin, but also contributes to determination of their quality.

It is used to control the quality level and classification of honeys of particular varieties and to detect changes in physicochemical and biological parameters. The method is dependent on the experience of the assessment team and must be supported by physicochemical tests. The result of the study depends on the experience of the research team [66,67,68,69,70,71,72].

This method is subjective and unreliable when examining less well-known honeys, because there is no reference point [65].

Exceeding the limit values of the sensory parameters.

The color, smell and taste of honey may depend on many factors, such as the origin of pollen, climate, weather conditions and storage time and conditions. Any change in these factors may result in organoleptic characteristics different than those standard for a given variety. The composition of the colored substances depends on the botanical origin of the honey and the place where the melliferous plants grow [54]. The content of aromatic substances decreases during heating and long storage [34,35].

Honey in its fresh and mature form should be a clear, highly hygroscopic liquid with a density of 1.38–1.45 g/cm^3^. The concentration of sugars (especially invert sugar and sucrose) affects the viscosity and density of honey in direct proportion [53].

Viscosity and crystal formation—crystallization is a natural process in honey. This process does not reduce the quality of the honey, but consumers prefer liquid honey. Glucose is responsible for the crystallization of honey, which is in a supersaturated state and therefore tends to reach equilibrium by crystallizing. Honeys with a predominance of fructose over glucose crystallize more slowly or not at all, e.g., acacia honeys. Fructose concentrates the solution, along with other sugars, and increases its viscosity, which makes it difficult for honey to crystallize. On the other hand, honeys with a predominance of glucose crystallize faster (rapeseed honey and dandelion honey). This parameter is not regulated by legal acts, but the very appearance of honey can indicate whether the honey is of the right variety and whether it has been heated [52].

### 2.3. Analysis of Physicochemical Parameters

The physicochemical parameters of honey quality are the basis for identifying the authenticity and adulteration of honeys.

The most useful parameter in identification is electrical conductivity. The use of this parameter makes it possible to distinguish nectar honeys of some varieties in relation to multifloral nectar honeys and, above all, the group of honeydew nectar honeys.

Other methods of differentiating honey varieties are as follows:

- Determination of the water content;
- Determination of the total and active acidity;
- Determination of the total ash content;
- Determination of the sugar content, including the ratio of glucose to fructose concentration (especially important when identifying heather honey);
- Analysis of aromatic acids and amino acids;
- Determination of the proline content;
- Determination of the diastase number;
- Determination of the proline content;
- Determination of the pH.

However, these methods used alone do not allow for the unambiguous differentiation of varietal honeys into particular types and varieties.

The methods that deserve special mention are as follows:

- Determination of the 5-HMF content;
- Determination of the diastase number.

These parameters make it possible to determine the level of honey aging and errors related to improper storage and thermal processing. The values of the above parameters change with the time of honey storage [61,69,70,71,72,73,74,75,76,77,78,79,80,81,82,83,84].

According to Popek [66], these methods cannot be considered fully reliable because the parameters change over time. These methods are time-consuming, cost-intensive, and their result does not provide unambiguous information about the authenticity of the honey. There are often problems with the interpretation and reproducibility of the results. Furthermore, the amount of reagent used to determine one sample has a negative impact on the environment.

### 2.4. Measurements of Color Parameters in L * a * b * and X Y Z Systems

The color of honey is one of the first features assessed by consumers.

Tristimulus colorimetry was instrumentally used to assess the color of honey. Color parameters L * a * b * were determined in the international CIE (Commission Internationale de l’Éclairage) system. The color is expressed in the CIE L * a * b * system, where L * is the lightness, and the a * and b * coordinates indicate the contribution of green (negative a * values), red (positive a *), blue (negative b * values) and yellow (b * values positive) [55,85,86,87].

This method requires properly prepared honey, which should be liquid with no signs of crystallization; otherwise, the test results may be different. The test is quick and easy to perform, but one should remember about the cost of purchasing the equipment and ensuring the repeatability of the test [85,86].

### 2.5. Extraction of Volatile Compounds

The suitability of the solid-phase microextraction (SPME) technique using gas chromatography coupled with mass spectrometry (GC-MS) is still being tested in determining the botanical authenticity of honeys. The qualitative analysis of volatile compound profiles is used to determine the botanical authenticity of nectar honeys [59,69,88,89,90,91,92,93,94,95,96,97].

### 2.6. Analysis of the Antioxidant Activity of Honey and Analysis of the Presence of Flavonoids

Polyphenolic compounds are among the most active antioxidants present in food. The use of spectral methods was aimed at determining the profiles of polyphenolic compounds, as well as assessing the antioxidant potential of individual varieties of bee honey. The botanical origin of honey significantly affects the antioxidant activity measured as the ability to scavenge DPPH• free radicals [75,98,99,100,101,102].

The use of the photochemiluminescence (PCL) test consists of the optical excitation of a UV sensitizer, which is responsible for generating free radicals, partially eliminated by the antioxidants present in the sample. Other radicals cause luminescence of the detected substance. The function of the sensitizer and detector is performed by the same compound, luminol. The measurement is fast and accurate as it only takes a few minutes to calculate based on the calibration curve, performed automatically by the software [84,103,104,105,106,107].

### 2.7. Nuclear Magnetic Resonance Spectroscopic Analysis

This analysis is very versatile, not only to assess the identification of the botanical origin of honey, but also to determine the composition and quality of the honey ingredients. For example, the diversity of honey components, including saccharides and all kinds of amino acids, is determined, which confirms their grouping according to the origin of the honey (using principal component analysis—PCA) [108,109,110,111,112,113,114,115,116,117]. 

Nuclear magnetic resonance (NMR) provides structural information with high re-producibility and accuracy. The time to obtain a 1H NMR spectrum is short (less than 5 min) and does not require calibration or standards. The advantage of the NMR method is the simultaneous detection of organic compounds in an unchanged state and conformation. The use of low-field 1 H NMR allows, based on increasing relaxation times, the detection of the addition of HFCS syrup in honey. The effective use of the NMR technique to identify honey adulteration is a very promising direction of research, but due to the relatively small number of reports, it is necessary to create an appropriate spectrum database, allowing for the quick interpretation of the results. Another factor limiting the use of NMR in analytics is the very high cost of the equipment [54].

### 2.8. Analysis of Honey Microscopic Image Identification

This is a method used when sweeteners are added to honey, e.g., sucrose by feeding bees or the adulteration of honey with the addition of sugar cane or fructose. In addition, this method shows the picture of honey impurities, e.g., nanoparticles or the presence/amount of yeast in the case of honeys with a high water content (e.g., added water). This test does not indicate unequivocal fermentation [118].

### 2.9. Analysis of the Isotopic Composition of Honey Using 13^C^/12^C^ Isotope-Ratio Mass Spectrometry Measurement

One of the spectrometric methods used to detect the adulteration of honey with the addition of cane or corn sugar and the incorrect declaration of the origin of honey is isotope-ratio mass spectrometry (IRMS) analysis. Isotope content is related to latitude, i.e., the climate prevailing in the place where the honey is obtained. This method is based on the use of proportions of isotopes characteristic of particular plant species. Its task is to estimate the amount or ratio of isotopes of one of the three basic elements (13^C^/12^C^, 180/16O, 2H/1H) and compare them with standard values [119,120,121,122,123].

This is a method with great potential, but it requires expensive equipment and specialized staff. In addition, its universal application does not guarantee the correctness of the results [124].

### 2.10. Chromatographic Analysis of Honey Composition

Chromatographic analysis using high-performance liquid chromatography (HPLC), gas chromatography (GC) and gas chromatography coupled with mass spectrometry (PTR-MS) is the basis for determining the composition and quality of bee honey. The results of these analyses are interpreted using statistical tools. The determination is quick, easy and effective, but costly. This method has many advantages: speed of measurement, low cost and use of a small amount of the test sample, which will not be destroyed. Rich libraries of spectra facilitate the identification of unknown substances. In the spectrum, different peaks may overlap, which can make interpretation difficult. In addition, the cost of purchasing equipment is high [125,126,127,128,129].

These methods allow for the differentiation of the botanical origin of monofloral and polyfloral honeys. However, HPLC shows an advantage over PTR-MS by providing much better differentiation of all analyzed types of honey. Chromatographic fingerprints recorded at 210 nm allow for the best classification of honey. Mass spectrometry with the proton transfer reaction is useful for distinguishing buckwheat honey [125,126,127,128,129].

### 2.11. Analysis of Glycerin or Ethanol Content

One of the methods used in assessing the authenticity (distinguishing natural from artificial honey) and freshness of honeys is the analysis of glycerin content. Glycerin is the result of metabolic processes caused by microorganisms present in the liquid collected by bees [130].

### 2.12. Fluorescence Spectroscopy Research

The advantage of fluorescence spectroscopy is the high sensitivity and specificity of classification. Fluorescence spectroscopy requires only minimal sample preparation. The results of the above studies confirmed that single synchronous fluorescence spectra of different honeys differ significantly due to their different physicochemical properties and provide sufficient data to clearly differentiate between groups of honeys. Studies have shown that this method is a valuable and promising technique for honey authentication. Honeys are well known to contain numerous fluorophores, such as polyphenols and amino acids. Some of them have been proposed as markers for monofloral honeys—ellagic acid for heather honey; hesperetin for citrus honey; phenylalanine and tyrosine, which turned out to be characteristic of lavender honey and made it possible to distinguish it from eucalyptus honey; and tryptophan and glutamic acid, which turned out to be useful for differentiating honeydew and flower honeys. Due to the presence of such powerful fluorophores, fluorescence spectroscopy can be helpful in confirming the botanical origin of honey.

In addition, these tests can be the basis for identifying honey overheating and identifying the botanical origin of filtered honeys, in which pollen analysis is not possible [131,132,133,134,135,136,137,138,139,140,141,142].

The limitation of this method is building a database of spectra characteristic for honeys. The method is fast, cheap and without a negative impact on the environment (no reagents are used) [139].

### 2.13. Infrared Spectroscopic Analysis

The authenticity of different types of honey can also be confirmed by infrared spectroscopy.

Infrared spectroscopy covers the spectrum of electromagnetic radiation in the range between the visible region and the microwave region (14,300–200 cm^−1^; 700–50,000 nm). Depending on the wavelength, it is divided into the following types of spectroscopy:

- Near infrared (NIR) spectroscopy, 14,300–4000 cm^−1^ (700–2500 nm);
- Mid (proper) infrared (MIR) spectroscopy, 700–4000 cm^−1^ (2500–14,300 nm);
- Far infrared (FIR) spectroscopy, 700–200 cm^−1^ (14,300–50,000 nm).

Quick quantitative and qualitative determination of the individual parameters that determine the quality of natural bee honeys is possible thanks to the use of spectroscopy in the NIR range. Through the basic analysis of the spectra, it is possible to distinguish between honeydew, artificial and nectar honeys, while using chemometrics, it is possible to determine the varieties of nectar honeys.

Studies conducted over the years have also shown the possibility of the presence of corn fructose in honey [78,79,127,133,143,144,145].

This method has many advantages: speed of measurement, low cost and use of a small amount of the test sample, which will not be destroyed. Rich libraries of spectra facilitate the identification of unknown substances. However, it should be remembered that different peaks in the spectrum may overlap, which can make interpretation difficult [143,144,145,146,147].

### 2.14. Research on Electrical Properties

The electrical properties of materials (impedance, permittivity and dielectric loss factor) describe the behavior of the material in an electric field. The molecular structure of the material is responsible for the physical and chemical properties, so there is a relationship between the electrical properties of a given material and its physical and chemical parameters. It is possible to use different dielectric quantities (electric permittivity, dielectric loss coefficient and conductivity) to differentiate honey varieties, and additives such as water and sugar are still being researched. In addition, these tests can also describe the level of honey overheating and the degree of crystallization [148,149,150,151,152,153,154,155,156].

### 2.15. Analysis of the Microbiological Purity of Honey

The examination of microbiological contamination of honey is aimed at assessing its quality. The parameters usually determined are coliform bacteria, sulfite-reducing *Clostridium*, yeasts and molds, aerobic mesophilic bacteria, *Salmonella* spp. and *Bacillus* spp. [73,113,152,153,156,157].

This method is time-consuming and not always effective. Its reproduction in the same conditions is impossible [73,156,157].

### 2.16. Research on Rheological Properties of Honeys

The crystal structure is a valuable source of information about honey. The rheological properties of honey indicate the characteristics of their origin and quality. The possibility of crystal formation and the observation of their behavior with the use of birefractive interferometry and computer image analysis can present the quantitative characteristics of the honey crystal structure used for its assessment. The analyses take into account standard stereological parameters, such as the number of identified objects and average values—surface area, circumference and maximum diameter of crystals. In addition, the numerical distribution of crystals with regard to the maximum diameter is analyzed. They can be used both to identify its origin and for other purposes, e.g., to determine the rheological properties of crystallized honey [157,158,159].

## 3. Conclusions

The authenticity of food products is rarely defined in the literature. However, there is some agreement in terms of nomenclature and a set of features that make it possible to recognize that a food product is what it should be, as declared by the manufacturer. Food adulteration is a frequent phenomenon, which should be considered a significant threat to every consumer—it is a violation of consumer rights, but also often leads to an increase in risk associated with the consumption of food products. Therefore, it is necessary to develop tools that will protect the consumer against abuse from dishonest producers.

Honey is often mentioned as an example of a product that can be counterfeited in various ways—its composition is changed (e.g., by adding sweeteners), and in recent times, consumers are often misled by giving false information about the geographical or botanical origin of honey. There are many research methods that are used to assess the authenticity of honeys, but above all, they ensure the confirmation of their quality. Regardless of the method adopted, the goal is always to determine whether the tested product is manufactured fairly and meets all legal criteria, or whether there has been a violation of the law. In the field of food authentication, there are targeted and non-targeted analytic methods that are proven by many studies [160]. For each of the products, a number of research methods can be indicated, but as in the case of assessing the quality and authenticity of honey, there is no combination of methods that would be 100% effective [54,161,162]. Currently, honey authenticity tests are difficult because not only local honeys are sold on domestic markets, but also foreign honeys from other continents, which are characterized by a different chemical composition and properties. Therefore, researchers are still looking for an ideal method that will not leave even 1% uncertainty in confirming the quality and authenticity of honeys [16]. The above list shows how active various researchers are in the search for a universal and relatively simple method of confirming the authenticity of honeys.

The present work provides a review not only of research methods, but also of their practical use. The methodological limitations and strengths of each method have been indicated. The work can be used as a resource and a quick path to finding an appropriate research method to determine the quality and authenticity of honeys.

In the example of honey quality assessment, it can be seen that single-component or multi-component analyses of honey quality parameters do not lead to obtaining unambiguous information on the botanical origin of honeys. However, they are helpful in more accurately indicating the place of origin and quality of honey. However, there is still no interdisciplinary, fast, effective and cheap method that can confirm the authenticity of honey, taking into account its quality characteristics.

The above analysis of the literature illustrates the pace at which the research and the methods used in the assessment of the quality of honey have developed. It demonstrates their differentiation, taking into account the aspects of varietal authenticity and the composition of honeys.

According to Kowalski and Łukasiewicz, the introduction of new, hitherto unknown techniques and the improvement of many measurement techniques (increasing their sensitivity and precision) are creating more and more tools and opportunities to identify falsifications, even at a very low level. However, it still requires an appropriate approach and experience from the analytical side, because honey is a product with a complicated analytical matrix [54]. This paper presents a review of the possible applications of methods with a high level of efficiency. Methods such as fluorescence, NIR and Raman spectroscopy seem to be multifaceted. However, they have limitations because they require a complex mathematical apparatus to interpret the results [54].

Summarizing this review of research methods, it was not possible to identify a method that is unequivocally the most effective. However, the analysis of research methods allowed us to identify 16 methodological groups in the field of honey quality and authenticity. The review of methods also allowed for the extraction of parameters indicating changes not only in the quality, but also in the authenticity of the honeys, along with the interpretation of exceeding the limit values of the parameters. The intention of the authors was to create a tool to help in selecting the most effective research methods, but also to combine several methods in order to obtain a reliable result.

## Figures and Tables

**Table 1:** Factors that define the quality of food products and the methods of their assessment.

Name	Parameters	Assessment Methods
Sensory and organoleptic attributes	- Color; - Taste; - Smell; - Texture; - Structure; - Appearance.	Objective measurements of physicochemical features responsible for shaping organoleptic features. Use of sensory panels carried out by a qualified research team.
Safety	Level of presence of toxic substances; organic and inorganic food additives; and microbiological, biochemical, chemical, physical and technological contamination.	Chemical, microbiological and biochemical analysis.
Health and nutritional value	The nutrient and non-nutrient content of the product and its energy value. In addition, it indicates the presence, assimilation and impact on the body of food additives, often with health-promoting effects, such as probiotics, polyphenolic compounds or vitamins.	Chemical and biochemical analysis of the composition of products and based on biological experiments.
Functional features	They mainly concern related aspects such as the ease of use of ingredients for processing, but also the size of the portions; in addition, their range is responsible for the characteristics of resistance to damage and storage stability.	Physical, biochemical and chemical analysis of raw materials and finished products.
Psychological parameters	The use of such features as convenience and ease of use at the appropriate price level and the level of novelty and attractiveness, taking into account the individual characteristics and needs of the consumer, makes the product habituated.	Market and consumer behavior research. The study of physiological reactions to stimuli and related behaviors

Source: [2,4,18,19,20].

**Table 2:** Physicochemical requirements for honeys, including the interpretation of exceeded parameters.

Parameter	Limit Value		Exceeding the Limit Values of the Parameters
Water content	Not more than 20%; however, not more than: (1) 23%—in heather honey and baker’s honey; (2) 25%—in heather baker’s honey.		The water content is considered as an indicator of honey stability and resistance to yeast fermentation. At a high level, it causes not only the fermentation and spoilage of honey, but also loss of taste. In addition, water activity is a parameter responsible for the growth of microorganisms [34,35]. In the case of changes in the water content, especially an increase, it can be presumed that water was added to the honey or it may indicate that the honey was removed from the hive too quickly. This parameter is also influenced by weather conditions during honey harvesting, e.g., the intensity of rainfall. An increase in its value is dangerous because it can affect the development of yeast and mold in honey [30,36]. Changes in this parameter may indicate adulteration of honey by adding, for example, invert sugar or potato (starch) syrup. It is added to honey to increase its weight. Adding water to honey has the same effect. This is very unfavorable for honey, as the increased amount of water increases the tendency to ferment [37].
Reducing sugar content (sum of fructose and glucose)	Not less than 60 g/100 g (nectar)	Not less than 45 g/100 g (nectar and honeydew)	The quantitative ratio of glucose to fructose is the main factor used to classify monofloral honeys [38]–acacia honey contains, on average, 34.6 g of fructose and 21.6 g of glucose; rapeseed honey, 37 g of fructose and 36.7 g of glucose; and dandelion honey, 35.9 g of fructose and 37.6 g of glucose [39,40]. It was observed that in honeydew honeys, the ratio of fructose to glucose content is higher than in nectar honeys. The only exception is black locust honey, in which the predominance of fructose over glucose ensures a liquid consistency for as long as several months. The preponderance of glucose is responsible for the rapid crystallization of honey, e.g., in rapeseed or dandelion honeys [38,39,40].
Sucrose content	Not more than 5 g/100 g, except when not more than: A total of 10 g/100 g—in locust (*Robinia pseudoacacia*), alfalfa (*Medicago sativa*), firewood banksia (*Banksia menziesii*), sweetvetch (*Hedysarum*), red rubber (*Eucalyptus camaldulensis*), leatherwood (*Eucryphia lucida*, *Eucryphia milligani*) and *Citrus* spp. honey; A total of 15 g/100 g—in lavender (*Lavandula* spp.) and borage (*Borago officinalis*) honey.		The content of saccharides in honey is determined, inter alia, by the origin of the honey, the time of harvesting and the length of the storage period; honeydew honeys contain more oligosaccharides and dextrins, while nectar honeys are dominated by simple sugars. Unripe honey contains the highest amount of sucrose. The content of individual carbohydrates may indicate the maturity of the honey or its lack [30,40]. An increased sucrose content may indicate adulteration of honey by feeding bees with sucrose or its addition to honey, but also the mixing varieties of honey [36]. Another very important indicator of honeydew honey is honeydew sugar—melezitose; this sugar is not found in nectar honeys. It may be an indicator of mixing honey varieties or a lack of varietal purity [27,30].
Free acid content	Not more than 50 mval/kg, but not more than 80 mval/kg in baking honey (industrial).		The value of the level of free acids—the level of free acids may indicate the maturity of the honey, as well as disorders related to the microbiological contamination of honey [35,41]. They can determine the taste and aroma of honey.
Diastase number (according to the Schade scale)	Not less than 8, except for baker’s (industrial) honey, but not less than 3 in honey with naturally low enzyme activity and an HMF content not more than 15 mg/kg.		The diastase number depends on the type and origin of the honey. Diastase (α-amylase) and invertase—enzymes derived from the salivary glands of bees—are among the most important biological components of honey. It is their presence that determines the nutritional and health-promoting properties of honey. The diastase number is an indicator of the enzymatic activity of honey. It is expressed by the number of Schade units per 1 g of honey. The level of diastase activity is one of the most important indicators that prove the high quality of honey. In the case of a low value of the diastase number, it can be presumed that the honey was heated to a temperature above 40 °C, which may also indicate the addition of sugar syrup [42] and the long storage of honey in unfavorable conditions [43,44].
5-hydroxymethylfurfural (HMF) content	Not more than 40 mg/kg, except for baker’s honey (industrial); also not more than 80 mg/kg in honey from regions with a tropical climate, and in mixtures of such honeys.		Content of 5-HMF (5-hydroxymethylfurfural)—this natural component of honey, heterocyclic 5-hydroxymethylfurfural aldehyde, is formed in an acidic environment from fructose (2-oxohexose). Natural honey does not contain 5-HMF or it is present in very small amounts (2–7 mg/kg). Its content may increase with prolonged storage and a too-high processing temperature; hence, 5-HMF is called the honey aging parameter. The content of 5-HMF proves not only the quality, but also the authenticity of the honey—the increased content of this ingredient also indicates adulteration with invert sugar or starch syrup. In the case of a very high level of this compound above 200 mg/kg, adulteration with chemical invert can be presumed [43,44,45,46]. HMF exhibits mutagenic activity and causes damage to the structure of the DNA helix [47]. HMF derivatives in the form of 5-sulfooxymethylfurfural (SOMF), 5-chloromethylfurfural and 5-hydroxymethyl-2-furancarboxylic acid (5-HMFK) have cytotoxic, genotoxic, neurotoxic, mutagenic and carcinogenic effects, which can lead to neoplastic changes in the liver, skin and lower colon tissues [42,48,49].
Proline content, mg/100 g of honey	Not less than 25 mg/100 g of honey.		Proline is an amino acid that is predominant in honey. In the case of the adulteration of natural honey with sucrose, a decrease in its content up to 10 mg/100 g of honey is observed [50]. A high proline content indicates the maturity of the honey; this indicator is often used in research as a honey quality parameter. The largest amounts of proline are found in buckwheat honey (approx. 80.8/100 g) [40].
Conductivity	Not more than 0.8 mS/cm, except for honeys and their mixtures listed below, but not less than 0.8 mS/cm in honeydew honey, chestnut honey and their mixtures. The conductivity of originating honey is not specified from strawberry tree (*Arbutus unedo*), heather (*Erica*), eucalyptus, linden (*Tilia* spp.), common heather (*Calluna vulgaris*), manuka leptospermum and tea tree (*Melaleuca* spp.)		The electrical conductivity of honey, as one of the physicochemical parameters, can be used to characterize its botanical origin, because it depends to a large extent on the plant from which honey was made [51,52]. The value of the electrical conductivity depends on the value of the level of mineral compounds and honey acids. In the case of this parameter, nectar honeys should have a value of up to 0.8 mS × cm^−1^, and honeydew honeys, due to a greater presence of minerals, should have this value above the level indicated above, even up to 1.5 times—up to 0.17 mS/cm^2^. Any deviations from this standard may indicate the mixing of nectar and honeydew honeys. The reduced value of electrical conductivity in honeydew honeys may indicate adulteration with nectar honey. Increased sucrose content combined with a reduced electrical conductivity, diastase number and proline content may indicate the adulteration of honey with sugar syrup [43,52].
Content of insoluble substances	No more than 0.1 g/100 g, but not more than 0.5 g/100 g in pressed honey		The content of insoluble substances indicates contamination of the hive or the product itself. The presence of these substances can lead to product contamination and consumer exposure [37,52].

Source: [11,24,26,53].

**Table 3:** Research methods used in assessing the quality and authenticity of varietal honeys.

Methods Used—Name	Applied Methods	Problem Identification Factors	Group of Honey Authenticity Problems
Melissopalynological analysis (honey pollen analysis)	Quantitative analysis consists of counting all plant parts (N), i.e., pollen grains, fungal spores and algae hyphae, yeast, starch grains and others in 10 g of honey. As a result, honey is assigned to one of five classes.	Identification of the leading pollen.	Identification of the botanical/geographical origin of the honey.
Sensory analysis	The bases for this form of research are the senses and feelings related to the smell, taste, color, appearance, and consistency of the product.	Identification of sensory features (color, taste, smell, appearance, consistency) characteristic of: - Varietal honeys, honey quality,—additives of prohibited substances; - Impurities - in honeys; - Signs of fermentation; - Consistency.	Identification of the botanical/geographical origin of the honey.
			Evaluation of the quality of bee honey.
			Counterfeit identification.
			Identification of bee honey fermentation.
			Identification of impurities.
Analysis of physicochemical parameters	The most useful parameters in identification are electrical conductivity, water content, total and active acidity, total ash content and sugar content, including the ratio of glucose to fructose concentration (especially important when identifying heather honey), the analysis of aromatic acids and amino acids, proline content, diastase number, proline content and pH level.	Identification of physicochemical parameters characteristic of: - Varietal honeys, - honey quality; - Additives of prohibited substances; - Impurities - in honeys; - Liquefied/heated/long-stored honeys.	Identification of the botanical/geographical origin of the honey.
			Evaluation of the quality of bee honey.
			Counterfeit identification.
			Identification of bee honey fermentation.
			Identification of impurities.
	The methods that deserve special mention are the determination of 5-HMF content and the determination of the diastase number.		Identification of heating/overheating of honey and improper storage conditions.
Measurements of color parameters in L * a * b * and X Y Z systems	Color parameters L * a * b * were determined in the international CIE system.	Identification of characteristic color parameters for varietal honeys.	Identification of too-long storage of honey.
Extraction of volatile compounds	The solid-phase microextraction (SPME) technique using gas chromatography coupled with a mass spectrometer (GC-MS).	Identification of volatile fractions of characteristic honeys for varietal honeys.	Identification of the botanical/geographical origin of the honey.
Analysis of antioxidant activity of honey and analysis of the presence of flavonoids	The botanical origin of honey significantly affects the antioxidant activity measured as the ability to scavenge DPPH• free radicals.	Identification of the level of antioxidant activity and the total value of characteristic polyphenols for given honey varieties.	Identification of the botanical/geographical origin of the honey.
			Evaluation of the quality of bee honey.
	The photochemiluminescence test (PCL).		Counterfeit identification.
NMR (nuclear magnetic resonance) spectroscopic analysis	This analysis is very versatile and is used with principal component analysis (PCA).	Identification and assessment of characteristic honey components for given honey varieties.	Identification of the botanical/geographical origin of the honey.
			Evaluation of the quality of bee honey.
	Metabolic analysis of organic extracts.		Counterfeit identification.
			Identification of impurities.
Analysis of honey microscopic image identification	This method shows the picture of honey.	Identification of additives and impurities.	Identification of honey adulteration with additives.
			Evaluation of the quality of bee honey.
			Identification of bee honey fermentation.
			Identification of impurities.
Analysis of the isotopic composition of honey using isotope ratio mass spectrometry (IRMS)	Measurement of the 13^C^/12^C^ isotope ratio.	Identification of additives and impurities.	Identification of honey adulteration with additives.
Chromatographic analysis of honey composition	Chromatographic analysis using high-performance liquid chromatography (HPLC), gas chromatography (GC) and gas chromatography coupled with mass spectrometry (PTR-MS).	Identification of additives and impurities.	Identification of honey adulteration with additives.
			Evaluation of the quality of bee honey.
			Identification of bee honey fermentation.
			Identification of impurities.
Analysis of glycerin or ethanol content	Analysis of glycerin content.	Identification of characteristics for fermented honeys.	Evaluation of honey quality.
			Identification of bee honey fermentation.
Fluorescence spectroscopy research	The advantage of fluorescence spectroscopy is the high sensitivity and specificity of classification.	Identification and assessment of honey authenticity.	Identification of the botanical/geographical origin of the honey.
Infrared spectroscopic analysis	Infrared spectroscopy covers the spectrum of electromagnetic radiation in the range between the visible region and the microwave region (14,300 and 200 cm^−1^; 700–50,000 nm).	Identification and assessment of honey authenticity.	Identification of honey adulteration with additives.
		Identification of ingredients determining the quality of natural bee honeys	Including, in particular, the adulteration of honey with sugar syrup from C4 plants. Identification of the botanical/geographical origin of the honey.
			Evaluation of the quality of bee honey.
Research on electrical properties	The electrical properties of materials (impedance, permittivity and dielectric loss factor) describe the behavior of the material in an electric field. The molecular structure of the material is responsible for the physical and chemical properties, so there is a relationship between the electrical properties of a given material and its physical and chemical parameters.	Identification and assessment of honey authenticity. Identification of characteristics for fermented honeys.	Identification of honey adulteration with additives.
			Evaluation of honey quality.
			Identification of the botanical/geographical origin of the honey.
Analysis of the microbiological purity of honey	The examination of the microbiological contamination of honey is aimed at assessing its quality; the parameters usually determined are coliform bacteria, sulfite-reducing *Clostridium*, yeasts and molds, aerobic mesophilic bacteria, *Salmonella* spp. and *Bacillus spp*.	Identification of characteristics for fermented honeys. Identification and assessment of honey authenticity.	Identification of honey adulteration with additives.
			Identification of the botanical/geographical origin of the honey.
Research on rheological properties of honeys	The crystal structure is a valuable source of information about honey. The rheological properties of honey indicate the characteristics of their origin and quality.	Identification and assessment of honey authenticity.	Identification of honey adulteration with additives.
			Evaluation of honey quality.

Source: Own research.

## Data Availability

No new data were created.

## References

[1] Haska A., Martyniuk E. (2019). Wybrane metody wyróżniania produktów spożywczych na rynku. Żywn. Nauka Technol. Jakość.

[2] Downey G. (2016). Advances in Food Authenticity Testing.

[3] (2015). Quality Management Systems—Requirements.

[4] Galanakis C.M. (2020). Innovative Food Analysis.

[5] Medina S., Pereira J.A., Silva P., Perestrelo R., Câmara J.S. (2019). Food fingerprints–A valuable tool to monitor food authenticity and safety. Food Chem..

[6] Cubero-Leon E., De Rudder O., Maquet A. (2018). Metabolomics for organic food authentication: Results from a long-term field study in carrots. Food Chem..

[7] Le Gall G., Puaud M., Colquhoun I.J. (2001). Discrimination between orange juice and pulp wash by 1H nuclear magnetic resonance spectroscopy: Identification of marker compounds. J. Agric. Food Chem..

[8] Rubert J., Lacina O., Zachariasova M., Hajslova J. (2016). Saffron authentication based on liquid chromatography high resolution tandem mass spectrometry and multivariate data analysis. Food Chem..

[9] Tena N., Aparicio-Ruiz R., Koidis A., García-González D.L. (2017). Analytical tools in authenticity and traceability of olive oil. Food Traceability and Authenticity.

[10] Spink J.W. (2019). Food Fraud Prevention: Introduction, Implementation and Management.

[11] Codex Standard for Honey, European Regional Standard CXS-12-1981, Codex Alimentarius, International Food Standards, Rev. 1. 1987, Rev. 2 2001. FAO, WHO, 2019, p. 52. www.fao.org/input/download/standards/310/cxs_012e.pdf.

[12] Śmiechowska M. (2007). Wybrane problemy autentyczności i identyfikowalności żywności ekologicznej. J. Res. Applic. Agric. Eng..

[13] Rozporządzenie Ministra Rolnictwa i Rozwoju Wsi z dnia 23 Grudnia 2014 r (2015). W Sprawie Znakowania Poszczególnych Rodzajów Środków Spożywczych.

[14] Rozporządzenie Ministra Rolnictwa i Rozwoju Wsi z dnia 29 maja 2015 r (2015). Zmieniające Rozporządzenie w Sprawie Szczegółowych Wymagań w Zakresie Jakości Handlowej Miodu.

[15] Spink J., Moyer D.C. (2011). Defining the public health threat of food fraud. J. Food Sci..

[16] Everstine K., Spink J., Kennedy S. (2013). Economically motivated adulteration (EMA) of food: Common characteristics of EMA incidents. J. Food Prot..

[17] Llano S.M., Muñoz-Jiménez A.M., Jiménez-Cartagena C., Londoño-Londoño J., Medina S. (2018). Untargeted metabolomics reveals specific withanolides and fatty acyl glycoside as tentative metabolites to differentiate organic and conventional Physalis peruviana fruits. Food Chem..

[18] Zawirska-Wojtasiak R., Jeleń H. (2012). Methods for sensory analysis. Food Flavours: Chemical, Sensory and Technological Properties.

[19] Wijayaa C.H., Wijaya W., Mehta B.M. (2015). General Properties of Major Food Components. Handbook of Food Chemistry.

[20] Piotrowska-Puchała A., Czernyszewicz E., Kołodziej E. (2018). Preferencje konsumentów, jakość i bezpieczeństwo nabywanej przez nich żywności. Jakość i Zarządzanie w Agrobiznesie Wybrane Aspekty.

[21] Niemczas-Dobrowolska M. (2021). Jakość i Bezpieczeństwo Żywności. Systemy, Postawy, Konsumenci.

[22] Grzybowska-Brzezińska M. (2018). Preferencje konsumentów wobec atrybutów produktów żywnościowych. Handel. Wewn..

[23] Tiwari K., Tudu B., Bandyopadhyay R., Chatterjee A., Pramanik P. (2018). Voltammetric sensor for electrochemical determination of the floral origin of honey based on a zinc oxide nanoparticle modified carbon paste electrode. J. Sens. Sens. Syst..

[24] (1988). Miód Pszczeli.

[25] Bogdanov S., Gallmann P. (2008). Authenticity of Honey and Other Bee Products: State of the Art.

[26] (2014). Council Directive 2014/63/EC of the European Parliament and of the Council of 15 May 2014 Amending Council Directive 2001/110/EC Relating to Honey.

[27] Guler A., Bakan A., Nisbet C., Yavuz O. (2007). Determination of important biochemical properties of honey to discriminate pure and adulterated honey with sucrose (*Saccharum officinarum* L.) syrup. Food Chem..

[28] Szczęsna T. (2003). Problemy z jakością miodu na rynku krajowym. Pasieka.

[29] Piotraszewska-Pająk A., Gliszczyńska-Świgło A. (2015). Directions of colour changes of nestar honeys depending on honey type and storage conditions. J. Apic. Sci..

[30] Soares S., Amaral J.S., Oliveira M.B.P.P., Mafra I. (2017). A Comprehensive review on the main honey. Authentication issues: Production and Origin. Compr. Rev. Food Sci. Food Saf..

[31] Dżugan M., Tomczyk M., Sowa P., Grabek-Lejko D. (2018). Antioxidant activity as biomarker of honey variety. Molecules.

[32] Kemsley E.K., Defernez M., Marini F. (2019). Multivariate statistics: Considerations and confidences in food authenticity problems. Food Control.

[33] (2001). Council Directive 2001/110/EC of 20 December 2001 Relating to Honey.

[34] Tornuk F., Karaman S., Ozturk I., Toker O.S., Tastemur B., Sagdic O., Kayacier A. (2013). Quality characterization of artisanal and retail Turkish blossom honeys: Determination of physicochemical, microbiological, bioactive properties and aroma profile. Ind. Crops Prod..

[35] Pentoś K., Łuczycka D., Wysoczański T. (2017). Dielectric properties of selected wood species in Poland. Wood Res..

[36] Laaroussi H., Bouddine T., Bakour M., Ousaaid D., Lyoussi B. (2020). Physicochemical Properties, Mineral Content, Antioxidant Activities, and Microbiological Quality of Bupleurum spinosum Gouan Honey from the Middle Atlas in Morocco. J. Food Qual..

[37] Wilczyńska A. (2012). Jakość Miodów w Aspekcie Czynników Wpływających na ich Właściwości Przeciwutleniające.

[38] Cianciosi D., Forbes-Hernández T.Y., Afrin S., Gasparrini M., Reboredo-Rodriguez P., Manna P.P., Battino M. (2018). Phenolic compounds in honey and their associated health benefits: A review. Molecules.

[39] Ruoff K., Luginbühl W., Kilchenmann V., Bosset J.O., von Der Ohe K., von Der Ohe W., Amadò R. (2007). Authentication of the botanical origin of honey using profiles of classical measurands and discriminant analysis. Apidologie.

[40] Jędrusek-Golińska A., Szymandera-Buszka K., Hęś M. (2023). Gospodarcze i prozdrowotne znaczenie miodu. Zag. Doradz. Rol..

[41] Terrab A., González A.G., Díez M.J., Heredia F.J. (2003). Characterisation of Moroccan unifloral honey using multivariate analysis. Eur. Food Res. Technol..

[42] Pasias I.N., Kiriakou I.K., Proestos C. (2017). HMF and diastase activity honeys: A fully validated approach and a chemometric analysis for identification of honey freshness and Adulteration. Food Chem..

[43] Kędzierska-Matysek M., Wolanciuk A., Florek M., Skałecki P., Litwińczuk A. (2017). Hydroxymethylfurfural content, diastase activity and colour of multifloral honeys in relation to origin and storage time. J. Cent. Europ. Agri..

[44] Wesołowska M., Dżugan M. (2017). Aktywność i stabilność termiczna diastazy występującej w podkarpackich miodach odmianowych. Żywn. Nauka Technol. Jakość.

[45] Al- Diab D., Jarkas B. (2015). Effect of storage and termal treatment on the quality of some locals brands of honey from Latakia markets. J. Entomol. Zool. Stud..

[46] Kursa K., Popek S. (2011). Próba zastosowania oceny poziomu 5-HMF jako wskaźnika jakości miodu pszczelego typu spadziowego. Zesz. Nauk. Uniw. Ekon. Poz..

[47] Teixido E., Nunez O., Santos F.J., Galceran M.T. (2011). 5-Hydroxymethylfurfural content in foodstuffs determined by micellar electrokinetic chromatography. Food Chem..

[48] Nikolov P.Y., Yaylayan V.A. (2011). Reversible and covalent binding of 5-(hydroxymethyl)-2-furaldehyde (HMF) with lysine and selected amino acids. J. Food Agric. Food Chem..

[49] Śliwińska A., Przybylska A., Bazylak G. (2012). Wpływ zmian temperatury przechowywania na zawartość 5-hydroksymetylofurfuralu w odmianowych i wielokwiatowych miodach pszczelich. Bromat. Chem. Toksykol.—XlV.

[50] Kursa K. (2015). Zawartość proliny jako wskaźnik autentyczności miodów. Zesz. Nauk. Akad. Morskiej Gdyni.

[51] Majewska E., Drużyńska B., Kowalska J., Wołosiak R., Ciecierska M., Derewiaka D. (2017). Zastosowanie metod fizykochemicznych i chemometrycznych do oceny jakości i autentyczności botanicznej miodów gryczanych. Zesz. Prob. Postępów Nauk Rol..

[52] Tichonow A.I., Bondarenko L.A., Jarnych T.G., Szpyczak O.S., Kowal W.M., Skrypnik–Tichonow R.I. (2017). Miód Naturalny w Medycynie i Farmacji (Pochodzenie, Właściwości, Zastosowanie, Preparaty Lecznicze).

[53] Zhu X., Li S., Zhang Z., Li G., Su D., Liu F. (2010). Detection of adulterants such as sweeteners materials in honey using near-infrared spectroscopy and chemometrics. J. Food Eng..

[54] Kowalski S., Łukasiewicz M. Zafałszowania i autentyczność miodu–metody identyfikacji. Proceedings of the Pszczoły Ludziom, Ludzie Pszczołom.

[55] Kuś P.M., Jerković I., Marijanović Z., Kranjac M., Tuberosod C.I.G. (2018). Unlocking Phacelia tanacetifolia Benth. honey characterization through melissopalynological analysis, color determination and volatiles chemical profiling. Food Res. Int..

[56] Flaczyk E., Górecka D., Korczak J. (2006). Towaroznawstwo Produktów Spożywczych.

[57] Louveaux J., Maurizio A., Vorwohl G. (1978). Methods of melissopalynology. Bee World.

[58] Thakodee T., Deowanish S., Duangmal K. (2018). Melissopalynological analysis of stingless bee (*Tetragonula pagdeni*) honey in Eastern Thailand. J. Asia-Pac. Entom.

[59] Karabagias I.K., Badeka A.V., Kontakos S., Karabournioti S., Kontominas M.G. (2014). Botanical discrimination of Greek unifloral honeys with physico-chemical and chemometric analyses. Food Chem..

[60] Puścion-Jakubik A., Brawska M.H. (2016). Odmianowe miody pszczele—pyłki główne i towarzyszące jako podstawa ich zaklasyfikowania. Probl. Hig. Epidemiol..

[61] Bodó A., Radványi L., Kőszegi T., Csepregi R., Nagya D.U., Ágnes F., Kocsis M. (2020). Melissopalynology, antioxidant activity and multielement analysis of two types of early spring honeys from Hungary. Food Biosci..

[62] Yang Y., Battesti M.-J., Gjabou N., Muselli A., Paolini J., Tomi P., Costa J. (2012). Melissopalynological origin determination and volatile composition analysis of Corsican “chestnut grove” honeys. Food Chem..

[63] Mureșan C.I., Cornea-Cipcigan M., Suharoschi R., Erler S., Mărgăoan R. (2022). Honey botanical origin and honey-specific protein pattern: Characterization of some European honeys. LWT.

[64] Serra Bonvehi J., Gomez Pajuelo A. (1988). Evaluation of honey by organoleptical analysis. Apiacta.

[65] Popek S. (2001). Studium Identyfikacji Miodów Odmianowych i Metodologii Oceny Właściwości Fizykochemicznych Determinujących ich Jakość.

[66] Kortesniemi M., Rosenvald S., Laaksonena O., Vanaga A., Ollikka T., Vene K., Yanga B. (2018). Sensory and chemical profiles of Finnish honeys of different botanical origins and consumer preferences. Food Chem..

[67] Vieira da Costa A.C., Batista Sousa J.M., Pereirada Silva M.A.A., dos Santos Garrut D., Madruga M.S. (2018). Sensory and volatile profiles of monofloral honeys produced by native stingless bees of the brazilian semiarid region. Food Res. Int..

[68] Rosiak E., Jaworska D. (2019). Właściwości probiotyczne i prebiotyczne miodów Pszczelich w aspekcie ich jakości i bezpieczeństwa zdrowotnego. Żywn. Nauka Technol. Jakość.

[69] Tischer Seraglio S.K., Bergamo G., Molognoni L., Daguer H., Silva B., Gonzaga L.V., Fett R., Oliviera Costa A.C. (2021). Quality changes during long-term storage of a peculiar Brazilian honeydew honey: “Bracatinga”. J. Food Comp. Anal..

[70] Srinual K., Intipunya P. (2009). Effects of crystallization and processing on sensory and physicochemical qualities of Thai sunflower honey. Asian J. Food Agro-Ind..

[71] Popek S. (1998). Electrical conductivity as an indicator of the quality of nectar honeys. Forum Ware.

[72] Popek S., Halagarda M., Kursa K. (2017). A new model to identify botanical origin of Polish honeys based on the physicochemical parameters and chemometric analysis. LWT.

[73] Mohamat R.N., Noor N.R.A.M., Yusof Y.A., Sabri S., Zawawi N. (2023). Differentiation of High-Fructose Corn Syrup Adulterated Kelulut Honey Using Physicochemical, Rheological, and Antibacterial Parameters. Foods.

[74] El Sohaimy S.A., Masry S.H.D., Shehata M.G. (2015). Physicochemical characteristics of honey from different origins. Ann. Agric. Sci..

[75] Chan B.K., Haron H., Talib R.A., Subramaniam P. (2017). Physical properties, antioxidant content and anti-oxidative activities of Malaysian stingless Kelulut (*Trigona* spp.). Honey J. Agric. Sci..

[76] Bakar M.A., Sanusi S.B., Bakar F.A., Cong O.J., Mian Z. (2017). Physicochemical and antioxidant potential of raw unprocessed honey from Malaysian stingless bees. Pak. J. Nutr..

[77] Bogdanov S., Ruoff K., Oddo L.P. (2004). Physico-chemical methods for the characterisation of unifloral honeys: A review. Apidologie.

[78] Acquarone C., Buera P., Elizalde B. (2007). Pattern of pH and electrical conductivity upon honey dilution as a complementary tool for discriminating geographical origin of honeys. Food Chem..

[79] Ruoff K., Luginbuhl W., Bogdanov S., Bosset J.O., Estermann B., Ziolko T., Amadò R. (2006). Authentication of the botanical origin of honey by near-infrared spectroscopy. J. Agric. Food Chem..

[80] Majewska E., Kowalska J. (2011). Badanie korelacji pomiędzy przewodnością elektryczną i zawartością popiołu w wybranych miodach pszczelich. Acta Agrophysica.

[81] Sykut B., Kowalik K., Hus W. (2018). Badanie jakości i zafałszowań miodów naturalnych. Postępy Tech. Przetw. Spoż..

[82] Mădaş N.M., Mărghitaş L.A., Dezmirean D.S., Bonta V., Bobiş O., Fauconnier M.-L., Francis F., Haubruge E., Nguyen K.B. (2019). Volatile Profile and Physico-Chemical Analysis of Acacia Honey for Geographical Origin and Nutritional Value Determination. Foods.

[83] Karabagias I.K., Vavoura M.V., Nikolaou C., Badeka A.V., Kontakos S., Kontominas M.G. (2014). Floral authentication of Greek unifloral honeys based on the combination of phenolic compounds, physicochemical parameters and chemometrics. Food Res. Int..

[84] Giemza M.A. (1999). Znaczenie Barwy w Ocenie Jakości Produktów na Przykładzie Miodów Odmianowych.

[85] Wilczyńska A. (2011). Wpływ procesów technologicznych na jakość miodów pszczelich—zmiany parametrów barwy oraz zawartości HMF pod wpływem przechowywania i ogrzewania. Zesz. Nauk. Uniw. Ekon. Pozn..

[86] Szabó R.T., Mézes M., Szalai T., Zajácz E., Weber M. (2016). Colour identification of honey and methodical development of its instrumental measuring. Columella. J. Agric. Environ. Sci..

[87] Radovic B.S., Careri M., Mangia A., Musci M., Gerboles M., Anklam E. (2001). Contribution of dynamic headspace GC-MS analysis of aroma compounds to authenticity testing of honey. Food Chem..

[88] Verzera A., Campisi S., Zappala M., Bonaccorsi I. (2001). SPME-GC/MS Analysis of honey volatile components for the characterization of different floral origin. Am. Lab..

[89] Majewska E., Delmanowicz A. (2007). Profile związków lotnych wybranych miodów pszczelich. Żywn. Nauka Technol. Jakość.

[90] Glory-Cuevas L.F. (2007). A review of volatile analytical methods for determining the botanical origin of honey. Food Chem..

[91] Jasicka-Misiak I., Kafarski P. (2011). Chemiczne markery miodów odmianowych. Wiad Chem..

[92] Majewska E. (2013). Studia nad Wykorzystaniem Wybranych Parametrów Fizyko-Chemicznych i Związków Lotnych do Określania Autentyczności Polskich Miodów Odmianowych.

[93] Escriche I., Sobrino-Gregorio L., Conchado A., Juan-Borrás M. (2017). Volatile profile in the accurate labelling of monofloral honey. The case of lavender and thyme honey. Food Chem..

[94] Morais da Silva P.L., de Lima L.S., Kaminski Caetano Í., Reyes Torres Y. (2017). Comparative analysis of the volatile composition of honeys from Brazilian stingless bees by static headspace GC–MS. Food Res. Int..

[95] Karabagias I.K., Badeka A.V., Kontominas M.G. (2020). A decisive strategy for monofloral honey authentication using analysis of volatile compounds and pattern recognition techniques. Microchem. J..

[96] Rodríguez-Flores M.S., Falcão S.I., Escuredo O., Seijo M.C., Vilas-Boas M. (2021). Description of the volatile fraction of Erica honey from the northwest of the Iberian Peninsula. Food Chem..

[97] Meda A., Euloga Lamiec C., Romito M., Millogo J., Nacoulma O. (2005). Determination of the total phenolic, flavonoid and proline contents in Burkina Fasan honey, as well as their radical scavenging activity. Food Chem..

[98] Wilczyńska A. (2010). Phenolic content and antioxidant activity of different types of polish honey—A short report. Pol. J. Food Nutr. Sci..

[99] Borawska M.H., Piekut J. (2006). Wartość liczby diastazowej, potencjał antyoksydacyjnego i zawartość polifenoli w miodach pszczelich z regionu Podlasia. Brom. Chem. Toksyk..

[100] Braghini F., Biluca F.C., Ottequir F., Gonzaga V.L., da Silva M., Vitali L., Micke G.A., Costa A.C.O., Fetta R. (2020). Effect of different storage conditions on physicochemical and bioactive characteristics of thermally processed stingless bee honeys. LWT.

[101] Da Silva P.M., Gonzaga L.V., Biluca F.C., Schulz M., Vitali L., Micke G.A., Costa A.C.O., Fetta R. (2020). Stability of Brazilian Apis mellifera L. honey during prolonged storage: Physicochemical parameters and bioactive compounds. LWT.

[102] Halagarda M., Groth S., Popek S., Rohn S., Pedan V. (2020). Antioxidant Activity and Phenolic Profile of Selected Organic and Conventional Honeys from Poland. Antioxidants.

[103] Popov I., Lewin G. (1999). Antioxidative homeostasis: Characterization by means of chemiluminescent technique. Methods Enzym..

[104] Dżugan M., Kisała J., Puchalski C., Bartosz G. (2011). Application of the Photochem system in agricultural investigations. Modern Methods in Analysis of Agricultural Raw Materials.

[105] Sarmento Silva T.M., Santos F.P., Evangelista-Rodrigues A., Sarmentoda Silva E.M., Sarmento da Silva G., Santosde Novais J., de Assis F., dos Santos R., Camaraa C.A. (2013). Phenolic compounds, melissopalynological, physicochemical analysis and antioxidant activity of jandaíra (*Melipona subnitida*) honey. J. Food Comp. Anal..

[106] Sidor A., Gramza-Michałowska A., Drgas M., Korczak J., Skręty J. (2013). Evaluation of chokeberry preparations antioxidant activity with use of the photochemilumiescence (PCL) assay. Probl. Hig. Epidemiol..

[107] Wesołowska M., Dżugan M. (2017). The use of the photochem device in evaluation of antioxidant activity of polish honey. Food Anal. Methods.

[108] Cazor A., Deborde C., Moing A., Rolin D., This H. (2006). Sucrose, glucose, and fructose extraction in aqueous carrot root extracts prepared at different temperatures by means of direct NMR measurements. J. Agric. Food Chem..

[109] Donarski J.A., Roberts D.P.T., Charlton A.J. (2010). Quantitative NMR spectroscopy for the rapid measurement of methylglyoxal in manuka honey. Anal. Methods.

[110] Boffo E.F., Tavares L.A., Tobias A.C.T., Ferreira M.M.C., Ferreira A.G. (2012). Identification of components of Brazilian honey by 1H NMR and classification of its botanical origin by chemometric methods. LWT Food Sci. Technol..

[111] Consonni R., Cagliani L.R., Cogliati C. (2012). NMR characterization of saccharides in Italian honeys of different floral sources. J. Agric. Food Chem..

[112] Zheng X., Zhao Y., Wu H., Dong J., Feng J. (2016). Origin Identification and Quantitative Analysis of Honeys by Nuclear Magnetic Resonance and Chemometric Techniques. Food Anal. Methods.

[113] Schievano E., Piana L., Tessari M. (2023). Automatic NMR-based protocol for assessment of honey authenticity. Food Chem..

[114] Chaji S., Olmo-García L., Serrano-García I., Carrasco-Pancorbo A., Bajoub A. (2021). Metabolomic approaches applied to food authentication: From data acquisition to biomarkers discovery. Food Authentication and Traceability.

[115] Zuccato V., Finotello C., Menegazzo I., Peccolo G., Schievano E. (2017). Entomological authentication of stingless bee honey by1H NMR-based metabolomics approach. Food Control.

[116] Schievano S., Stocchero M., Zuccato V., Conti I., Piana L. (2019). NMR assessment of European acacia honey origin and composition of EU-blend based on geographical floral markers. Food Chem..

[117] Gerginova D., Simova S., Popova M., Stefova M., Stanoeva J.P., Bankova V. (2020). NMR profiling of North Macedonian and Bulgarian honeys for detection of botanical and geographical origin. Molecules.

[118] Kerkvliet J.D., Meijer H.A.J. (2000). Adulteration of honey: Relation between microscopic analysis and delta C-13 easurements. Apidologie.

[119] Przetaczek-Rożnowska I., Rosiak M. (2011). Wykrywanie zafałszowań żywności. Przem. Spoż.

[120] Lipiński Z., Czajkowska K. (2012). Metody nowoczesnego wykrywania zafałszowań miodu syropami cukrowymi ze skrobi, Higiena żywności i pasz. Życie Wet..

[121] She S., Chen L., Song H., Lin G., Li Y., Zhou J., Liu C. (2019). Discrimination of geographical origins of Chinese acacia honey using complex 13C/12C, oligosaccharides and polyphenols. Food Chem..

[122] Bong J., Middleditch M., Stephens J.M., Loomes K.M. (2023). Analiza proteomiczna miodu: Profilowanie peptydów jako nowatorskie podejście do uwierzytelniania miodu Mānuka (*Leptospermum scoparium*) w Nowej Zelandii. Żywność.

[123] Wang X., Yan S., Zhao W., Wu L., Tian W., Xue X. (2023). Comprehensive study of volatile compounds of rare Leucosceptrum canum Smith honey: Aroma profiling and characteristic compound screening via GC–MS and GC–MS/MS. Food Res. Int..

[124] Yu W., Zhang G., Wu D., Guo L., Huang X., Ning F., Luo L. (2023). Identification of the botanical origins of honey based on nanoliter electrospray ionization mass spectrometry. Food Chem..

[125] Cabanero A.I., Recio J.L., Ruperez M. (2006). Liquid chromatography coupled to isotope ratio mass spectrometry: A new perspective on honey adulteration detection. J. Agric. Food Chem..

[126] Kuś P.M., van Ruth S. (2015). Discrimination of Polish unifloral honeys using overall PTR-MS and HPLC fingerprints combined with chemometrics. LWT–Food Sci. Technol..

[127] Nasaba S.G., Yazd M., Marini F., Nescatelli R., Biancolillo A. (2020). Classification of honey applying high performance liquid chromatography, near-infrared spectroscopy and chemometrics. Chemom. Intell. Lab. Syst..

[128] Wang Y., Xing L., Zhang J., Ma X., Weng R. (2023). Determination of endogenous phenolic compounds in honey by HPLC-MS/MS. LWT.

[129] Beckh G., Wessel P., Lullmann C. (2005). Contribution to yeasts and their metabolisms products as natural components of honey—Part 3: Contents of ethanol and glycerol as quality parameters. Dtsch. Leb. Rundsch.

[130] Zucchi P., Marcazzan G.L., Dal Pozzo M., Sabatini A.G., Desalvo F., Floris I. (2006). Il contenuto di etanolo nel miele per la valutazione di processi fermentativi. APOidea.

[131] Gębala S. (2009). Measurements of solution fluorescence—A new concept. Opt. Appl..

[132] Karoui R., Dufour E., Bosset J.-O., De Baerdemaeker J. (2007). The use of front face fluorescence spectroscopy to classify the botanical origin of honey samples produced in Switzerland. Food Chem..

[133] Lenhardt L., Bro R., Zekovic I., Dramicanin T., Dramicanin M.D. (2015). Fluorescence spectroscopy coupled with PARAFAC and PLS DA for characterization and classification of honey. Food Chem..

[134] Lenhardt L., Zekovic I., Dramicanin T., Dramicanin M.D., Bro R. (2014). Determination of the botanical origin of honey by front-face synchronous fluorescence spectroscopy. Appl. Spectrosc..

[135] Dramićanin T., Lenhardt L., Ackovic L., Zekovic I., Dramicanin M.D. (2018). Detection of Adulterated Honey by Fluorescence Excitation-Emission Matrices. J. Spectrosc..

[136] Wilczyńska A., Żak N. (2020). The Use of Fluorescence Spectrometry to Determine the Botanical Origin of Filtered Honeys. Molecules.

[137] Parri E., Santinami G., Domenici V. (2020). Front-Face Fluorescence of Honey of Different Botanic Origin: A Case Study from Tuscany (Italy). Appl. Sci..

[138] Xagoraris M., Revelou P.-K., Alissandrakis E., Tarantilis P.A., Pappas C.S. (2021). The Use of Right Angle Fluorescence Spectroscopy to Distinguish the Botanical Origin of Greek Common Honey Varieties. Appl. Sci..

[139] Żak N., Wilczyńska A., Przybyłowski P. (2018). Zastosowanie spektroskopii fluoroscencyjnej do oceny stopnia podgrzania miodu. Folia Pomeranae Univ. Technol. Stetin..

[140] Truong H.T.D., Reddy P., Reis M.M., Archer R. (2023). Internal reflectance cell fluorescence measurement combined with multi-way analysis to detect fluorescence signatures of undiluted honeys and a fusion of fluorescence and NIR to enhance predictability. Spectrochim. Acta Part A Mol. Biomol. Spectrosc..

[141] Ruoff K., Iglesias M.T., Luginbuehl W., Jacques-Olivier B., Stefan B., Amado R. (2005). Quantitative analysis of physical and cemical measurands in honey by mid-infrared spectrometry. Eur. Food Res. Technol..

[142] Ruoff K., Karoui R., Dufour E., Luginbuhl W., Bosset J.O., Bogdanov S., Amadò R. (2005). Authentication of the botanical origin of honey by front-face fluorescence spectroscopy, a preliminary study. J. Agric. Food Chem..

[143] Ruoff K., Luginbühl W., Künzli R., Bogdanov S., Bosset J.O., von der Ohe K., von der Ohe W., Amadò R. (2006). Authentication of the botanical and geographical origin of honey by front-face fluorescence spectroscopy. J. Agric. Food Chem..

[144] Kelly J.D., Petisco C., Downey G. (2006). Potential of near infrared transflectance spectroscopy to detect adulteration of Irish honey by beet invert syrup and high fructose corn syrup. J. Near Infrared Spectr..

[145] Woodcock T., Downey G., O’Donnell C.O. (2009). Near infrared spectral fingerprinting for confirmation of claimes PDO provenance of honey. Food Chem..

[146] Szterk A., Lewicki P. (2010). Spektroskopia NIR on-line w kontroli procesów produkcji żywności. Przem. Spoż.

[147] Piekut J. (2011). Zastosowanie Spektroskopii w Bliskiej Podczerwieni (NIR) Do Analizy Wybranych Parametrów Jakościowych Naturalnych Miodów Pszczelich.

[148] Łuczycka D. (2010). Właściwości dielektryczne wybranych odmian miodu. Inżynieria Rol..

[149] Pentoś K., Łuczycka D., Kapłon T. (2015). The identification of relationships between selected honey parameters by extracting the contribution of independent variables in a neural network model. Eur. Food Res. Technol..

[150] Pentoś K., Łuczycka D., Wróbel R. (2014). The identification of the relationship between chemical and electrical parameters of honeys using artificial neural networks. Comp. Biol. Med..

[151] Łuczycka D., Pentoś K. (2019). The use of dielectric honey features for overheating diagnostics. Acta Aliment..

[152] Łuczycka D., Pentoś K., Wysoczański T. (2016). The influence of crystallization and temperature on electrical parameters of honey. Zesz. Probl. Postępów Nauk Rol..

[153] Vozáry E., Ignáczk K., Gillay B. (2020). Dielectrical properties of Hungarian acacia honeys. Prog. Agric. Eng. Sci..

[154] Pentoś K. (2016). The methods of extracting the contribution of variables in artificial neural network models—Comparison of inherent instability. Comp. Electr. Agric..

[155] Pentoś K., Łuczycka D. (2018). Dielectric properties of honey—The potential usability for quality assessment. Eur. Food Res. Technol..

[156] Bakier S. (2008). Badania Właściwości Reologicznych Miodu w Postaci Skrystalizowanej.

[157] Addo M.G., Mutala A.H., Badu K. (2020). A Comparative Study on the Antimicrobial Activity of Natural and Artificial (Adulterated) Honey Produced in Some Localities in Ghana. Int. J. Curr. Microbiol. App. Sci..

[158] Yilmaz M.T., Tatlisu N.B., Toker O.S., Karaman S., Dertli E., Sagdic O., Arici M. (2014). Steady, dynamic and creep rheological analysis as a novel approach to detect honey adulteration by fructose and saccharose syrups: Correlations with HPLC-RID results. Food Res. Int..

[159] Kamboj U., Mishra S. (2015). Prediction of adulteration in honey using rheological parameters. Int. J. Food Prop..

[160] Karoui R. (2020). Food authenticity and fraud. Chemical Analysis of Food.

[161] Brar D.S., Pant K., Krishnan R., Kaur S., Rasane P., Nanda V., Gautam S. (2023). A comprehensive review on unethical honey: Validation by emerging techniques. Food Control.

[162] Valverde S., Ares A.M., Elmore J.S., Bernal J. (2022). Recent trends in the analysis of honey constituents. Food Chem..

